# Annexin-A1 Tripeptide Attenuates Surgery-Induced Neuroinflammation and Memory Deficits Through Regulation the NLRP3 Inflammasome

**DOI:** 10.3389/fimmu.2022.856254

**Published:** 2022-05-06

**Authors:** Zhiquan Zhang, Qing Ma, Ravikanth Velagapudi, William E. Barclay, Ramona M. Rodriguiz, William C. Wetsel, Ting Yang, Mari L. Shinohara, Niccolò Terrando

**Affiliations:** ^1^Department of Anesthesiology, Duke University Medical Center, Durham, NC, United States; ^2^Department of Immunology, Duke University Medical Center, Durham, NC, United States; ^3^Department of Psychiatry and Behavioral Sciences, Mouse Behavioral and Neuroendocrine Analysis Core Facility, Duke University Medical Center, Durham, NC, United States; ^4^Department of Neurobiology, Duke University Medical Center, Durham, NC, United States; ^5^Department of Cell Biology, Duke University Medical Center, Durham, NC, United States; ^6^Department of Medicine, Division of Nephrology, Duke University Medical Center, Durham, NC, United States; ^7^Department of Molecular Genetics and Microbiology, Duke University Medical Center, Durham, NC, United States

**Keywords:** NLRP3 inflammasome, postoperative cognition dysfunction, microglia, annexin A1 derived peptide, aging

## Abstract

Neuroinflammation is a growing hallmark of perioperative neurocognitive disorders (PNDs), including delirium and longer-lasting cognitive deficits. We have developed a clinically relevant orthopedic mouse model to study the impact of a common surgical procedure on the vulnerable brain. The mechanism underlying PNDs remains unknown. Here we evaluated the impact of surgical trauma on the NLRP3 inflammasome signaling, including the expression of apoptosis-associated speck-like protein containing a CARD (ASC), caspase-1, and IL-1β in the hippocampus of C57BL6/J male mice, adult (3-months) and aged (>18-months). Surgery triggered ASC specks formation in CA1 hippocampal microglia, but without inducing significant morphological changes in NLRP3 and ASC knockout mice. Since no therapies are currently available to treat PNDs, we assessed the neuroprotective effects of a biomimetic peptide derived from the endogenous inflammation-ending molecule, Annexin-A1 (ANXA1). We found that this peptide (ANXA1sp) inhibited postoperative NLRP3 inflammasome activation and prevented microglial activation in the hippocampus, reducing PND-like memory deficits. Together our results reveal a previously under-recognized role of hippocampal ANXA1 and NLRP3 inflammasome dysregulation in triggering postoperative neuroinflammation, offering a new target for advancing treatment of PNDs through the resolution of inflammation.

## Introduction

Perioperative neurocognitive disorders (PNDs), which include acute delirium and lingering cognitive impairment following surgery and hospitalization, are common especially within the rapidly growing senior population (1). These neurologic complications are significant problems that can profoundly alter recovery from routine procedures such as orthopedic surgery (2). Aside from the implications of a worsened perioperative course that include prolonged hospital length-of-stay, higher rates of nursing home placement, and soaring healthcare costs, PNDs lead to a persistent functional decline, including dementia, and they also impact mortality rates (3, 4). Although some strategies, such as unit-based targeted multifactorial intervention and proactive geriatric consultation have been implemented to prevent postoperative delirium (5), no Food and Drug Administration approved drug is currently available to treat PNDs

Aberrant inflammation is generally considered to be a driver of PNDs (6). We have developed a clinically relevant mouse model of PNDs by using a tibial fracture method that mimics common aspects of orthopedic trauma and repair, and we have studied its effects on the central nervous system (CNS) (7–9). Using this model, we have demonstrated a role for systemic cytokines that promote neuroinflammation and glial activation in the hippocampus (7, 8). Anti-inflammatory agents can ameliorate this cytokine storm and thereby prevent neuroinflammation and the presentation of PND-like behaviors in rodents. However, the possibility of side effects that involve overt immunosuppression, raises concerns for clinical translation (10). Resolution pharmacology has provided a shift in the approach to treat inflammatory conditions by focusing on stimulating pro-resolving pathways vs. simply blocking pro-inflammatory molecules (11). To date several endogenous pro-resolving pathways have been identified and these include bioactive lipids, autacoids, and peptides (e.g., annexin-A1 or ANXA1) (12). These bioactive mediators are implicated in arthritis, diabetes, stroke, dementia, and other inflammatory conditions, yet little is known about their mechanisms of action and targets in PNDs.

ANXA1 is a glucocorticoid-regulated protein with well-established effects on the immune system, that involves regulation of leukocyte trafficking, endothelial integrity, and microglial activation (13). Several peptides (Ac2-26, Ac2-12, and Ac2-6) have been derived from the full-length N-terminal domain of ANXA1 and they have been tested in several inflammatory conditions (14). We have developed a small peptide (ANXA1sp) with potent anti-inflammatory effects on NF-κB signaling and organ-preserving functions (15–17). Based on preclinical and clinical results on the involvement of interleukin (IL)-1β signaling in PNDs (7, 18), we have hypothesized that NLRP3 inflammasome activation orchestrates postoperative neuroinflammation. Here, we have tested the ability of ANXA1sp to modulate NLRP3 inflammasome activation. Our data show that orthopedic surgery downregulates ANXA expression in the hippocampus, which can be restored by prophylaxis with ANXA1sp both in adult and aged mice. Further, we observe that surgery activates the NLRP3 inflammasome complex, including ASC speck formation in hippocampal microglial, which may control the subsequent development of PND-related behavior. Notably, administration of ANXA1sp reduces NLRP3 inflammasome activation across ages. Reduction of the postoperative neuroinflammation resulted in overall improvement of hippocampal-dependent memory deficits.

## Materials and Methods

### Animals

All experimental procedures were conducted under protocols approved by the Institutional Animal Care and Use Committee at Duke University in accordance with the guidelines of the National Institutes of Health for animal care (Guide for the Care and Use of Laboratory Animals, Health and Human Services, National Institutes of Health Publication No. 86-23, revised 1996). Studies were conducted on C57BL6/J male adult mice (3 months; weight 20-26 g) and aged mice (18-22 months; 26-55 g) (Jackson Laboratories, Bar Harbor, ME). *Nlrp3^-/-^* (19), *Asc^-/-^* (19), and ASC-citrine mice (20) (3-4 months; weight 20-26 g) were bred in house. Mice were housed (3-5 animals/cage) in a 12:12-h light:dark cycle in a humidity- and temperature-controlled room with free access to food and water. The animals were acclimated for at least 1 week before starting any experiment. Note, in the aged cohort, 14 mice were excluded due to complications after surgery (tumor, stroke, death), while no mice from any other cohort were excluded.

### Drug Treatments

Peptide preparation and treatment were performed as previously described (16). Briefly, ANXA1 biomimetic tripeptide (ANXA1sp, Ac-QAW, or QW-3 (15); Ac = acetyl, MW = 445.47 Da) was synthesized and purified (> 98% purity) by GenScript (Piscataway, NJ). The tripeptide was suspended in 100% DMSO and diluted in saline to a final dose of 1 mg/kg ANXA1sp in 0.5% DMSO. Mice were injected intraperitoneally with 1 mg/kg ANXA1sp in 0.5% DMSO and saline 30 min before orthopedic surgery.

### Orthopedic Surgery and Sample Preparation

We performed an open tibial fracture as previously described (21). Briefly, mice were injected with 0.1 mg/kg buprenorphine (s.c.) and they underwent orthopedic surgery with 2.1% isoflurane anesthesia. Mice not exposed to surgical manipulations served as naïve controls. Mice were euthanized under isoflurane anesthesia and were perfused with ice-cold PBS to flush blood from the brain vasculature. Hippocampi were rapidly (< 2 min) dissected on ice, frozen in liquid nitrogen for protein assays, and stored at -80°C until assay.

Mice destined for immunostaining were perfused with an ice-cold solution of 4% paraformaldehyde (PFA) PBS solution. Whole brains were incubated at 4°C in the 4% PFA solution overnight. The next day brains were rinsed in ice-cold PBS and incubated in 20% and 30% sucrose at 4°C for 24 h, respectively. The brains were placed into OCT solution and stored at -80°C for further use.

### Western Blot

Mouse hippocampi were lysed in radioimmunoprecipitation assay (RIPA) buffer (R0278; Sigma) containing Halt Protease Inhibitors Cocktail (78430; Thermo Scientific). Proteins were separated on 4%-15% or 4%-20% Criterion TGX precast protein gels (64134751; Bio-Rad), and transferred to an Immun-Blot PVDF membrane (1620177; Bio-Rad). After blocking with 5% nonfat dry milk (170-6404; Bio-Rad), the blot was probed with the following antibodies (1:1000): NLRP3 (LS-C374964; LSBio), ASC (67824S; Cell Signaling), Caspase1 (Cleaved Asp210) (LS-C380449; LSBio), IL-1β (LS-C104813; LSBio), and Annexin-A1 (ANXA1) (3299S; Cell Signaling) or (71-3400; Invitrogen). The proteins were visualized using SuperSignal West Dura Extended Duration Substrate (34076; Thermo Scientific) on a ChemiDoc MP Imaging System (Bio-Rad). Protein loading was assessed using anti-GAPDH (2118S; Cell Signaling). Bands were quantified with Fiji software, and analyzed with Prism 8 (GraphPad Software). Hippocampal levels of ATP were determined using a commercially available assay kit (ab83355, Colorimetric/Fluorometric) following the manufacturer’s instructions.

### Immunofluorescence

Immunostaining and analyses of microglial morphology were performed on the PFA-perfused slices using ionizing calcium-binding adaptor molecule 1 (Iba1) rabbit antibody (019-19741; Wako). Briefly, brain slices (30 μm thick) were incubated in 10% normal donkey or goat serum in PBST [1 x PBS (11189, Gibco) + 0.5% Tween 20 (P1379, Sigma)] containing 0.3% Triton X 100 (T8787, Sigma) for antigen retrieval and to block any background for 2 h at RT. Slices were incubated with the primary antibody (1:500) at 4°C overnight. After 3 washes with PBST, slices were incubated with the secondary antibody conjugated with Alexa Fluor 488 or Fluor 647 (1:500, Invitrogen) for 2 h at RT. After 5 washes with PBST, the sections were mounted, dehydrated, and cover-slipped. Images were acquired using Zeiss 880 inverted confocal Airy scan.

### Image Analyses

Imaged tile scans were stitched together and presented as high-resolution images. Microglial morphology was quantified as described in Schwarz et al. (22), based on 4 morphologic subtypes: round/amoeboid microglia (*Round*), stout microglia (*Stout*), microglia with thick long processes (*Thick*), and microglia with thin ramified processes (*Thin*). The number of Iba1-positive cells was determined in three representative areas of the hippocampal CA1 region per animal. To determine the state of microglial activation, we manually traced their branch lengths using Imaris Filament Tracer to compare branch lengths from soma and the complexity of individual microglia in the CA1 region before and after orthopedic surgery.

### Behavioral Assays: “What-Where-When” Memory and Memory Load

Hippocampal-dependent memory was assessed with two mnemonic tasks (23, 24). The first task assessed memory for objects (“what”), their locations (“where”), and the order in which they were presented (“when”). A second task examined memory load with seven unique objects. For the What-Where-When (W-W-W) task, training/testing were conducted in a 65 x 52 x 25 cm arena with complex visual cues on the walls of the room. Briefly, mice were acclimated to the arena for 5 min and then were subjected to two 5-min training and a single test trial, each separated by 55-60 min. In the first training trial, mice were presented with 4 identical objects (Set A). Three of the objects were spaced equally along the long axis of one wall with the fourth object centered along the opposite wall in a triangle formation. In the second training trial (Set B), a new set of 4 identical objects was presented. Objects 1 and 2 of Set B overlapped in location with objects 1 and 3 of Set A, while the two remaining objects of Set B were positioned in corners 3 and 4. At testing two objects each from Sets A and B were used, for a total of 4 objects. One of the Set A objects was placed in corner 1 (stationary object A). The second Set A object was placed in corner 3 (displaced Set B object). Set B objects remained in corners 2 and 4 (“recent” objects). Behavior was filmed for all trials and scored by blinded researchers subsequently with Noldus Ethovision 11 (Noldus Information Technologies, Asheville, NC) using nose-point tracking. An object interaction was scored when the nose point was within 2 cm of the object center with the body axis of the mouse oriented toward the object. All preference scores were calculated from the test trial. “What” preference was calculated as the time exploring the two A objects subtracted from the exploration times of the two B objects (recent objects in corners 2 and 4), divided by the total times for all of the A and B objects. “When” memory preference was calculated as the exploration time for stationary A object (in corner 1) minus the exploration times of the two B objects (corners 2 and 4), divided by the total times with these three objects. “Where” preference score was calculated as the difference between the exploration time for the A object (which displaced object B in corner 3) minus the time with stationary A object (corner 1) divided by the total times with these two objects.

Twenty-four hours after completion of the W-W-W task, the mice were subjected to a seven-trial memory load test (23, 24). This test was conducted in the same arena as the W-W-W task, but without visual cues on the walls. Testing was performed over 7 consecutive test trials, with an approximate 60-90 sec inter-trial interval. To accommodate the increasing number of objects on each trial, the first three trials were 3 min, trials 4-5 were 4 min, and trials 6 -7 were 5 min in length. On trial 1, one object was presented. With each subsequent trial a novel object (i.e., differing in configuration, color, and texture) was added in a random location to prevent the animal from predicting the new object based on the previous object’s placement. By the end of testing, all objects were equally spaced with no object being within 8-10 cm from the nearest object or wall of the test arena. Once the exploration time for a given trial is over, the mouse was placed into an empty mouse cage while the next object was placed in the arena before the mouse was reintroduced into the arena in the same location as before. Testing was filmed and scored using Noldus Ethovision 11 as in the W-W-W test. Exploration time with test objects was expressed as time spent in contact with the object/min to adjust for the varying lengths of the test-trials. Preference for the new object in each trial was expressed as the time spent with the new object minus the time spent with the remaining objects, divided by the sum of these times. The preference ratio ranged from -1 to +1, where positive scores indicated a preference for the new object, negative scores indicated a preference for the previously introduced objects, and a score approaching “0” indicated no preference.

### Statistical Analysis

The behavioral data were analyzed with SPSS 25 (IBM SPSS, Chicago, IL), while all other data were analyzed with Prism 8 (GraphPad Software). The preference scores and time interacting with objects were analyzed with multivariate ANOVA (MANOVA). In the W-W-W test, the dependent variables were for the 3 different preference scores that were assessed simultaneously at testing. In the memory load test, the six different preference scores for trials 2-7 were the dependent variables. In all analyses the treatment condition was the fixed factor. *Post-hoc* tests were with Tukey corrected pair-wise comparisons. The statistics for the behavioral studies are in **Tables 1**–**4**. All non-behavioral data were analyzed by t-tests or ANOVA followed by Tukey’s multiple comparisons *post-hoc* tests. These data are presented as mean ± SEM. In all cases, a *P*<0.05 was considered statistically significant.

**Table 1:** MANOVA results for “What”, “Where”, and “When” preference scores.

Test^a^	Degrees of freedom	F-score	P Value
What	2,27	2.762	0.081
When	2,27	5.845	0.008
Where	2,27	5.560	0.010

a N=10 mice/group (naïve, vehicle + surgery, ANXA1sp + surgery).

**Table 2:** MANOVA results for “What”, “Where”, and “When” time spent exploring objects^a^.

Test^b^	Degrees of freedom	F-score	P Value
Set A objects	2,27	1.877	0.173
Set B objects	2,27	5.391	0.011
Test	2,27	18.732	0.001

a Analysis is across the two training sessions with Set A and Set B objects and at testing.

b N=10 mice/group (naïve, vehicle + surgery, ANXA1sp + surgery).

**Table 3:** MANOVA results for memory load preference scores.

Test^a^	Degrees of Freedom	F-score	P Value
2 Objects	2,27	2.956	0.053
3 Objects	2,27	4.462	0.021
4 Objects	2,27	8.143	0.002
5 Objects	2,27	25.427	0.001
6 Objects	2,27	12.434	0.001
7 Objects	2,27	2.913	0.057

a N=10 mice/group (naïve, vehicle + surgery, ANXA1sp + surgery).

**Table 4:** MANOVA results for memory load time spent exploring objects.

Test^a^	Degrees of freedom	F-score	P Value
2 Objects	2,27	17.236	0.003
3 Objects	2,27	0.327	0.724
4 Objects	2,27	1.464	0.249
5 Objects	2,27	3.789	0.035
6 Objects	2,27	8.310	0.002
7 Objects	2,27	2.077	0.145

a N=10 mice/group (naïve, vehicle + surgery, ANXA1sp + surgery).

## Results

### Postoperative Resolution of CNS Inflammation and NLRP3 Inflammasome Activation

**Figure 1:**
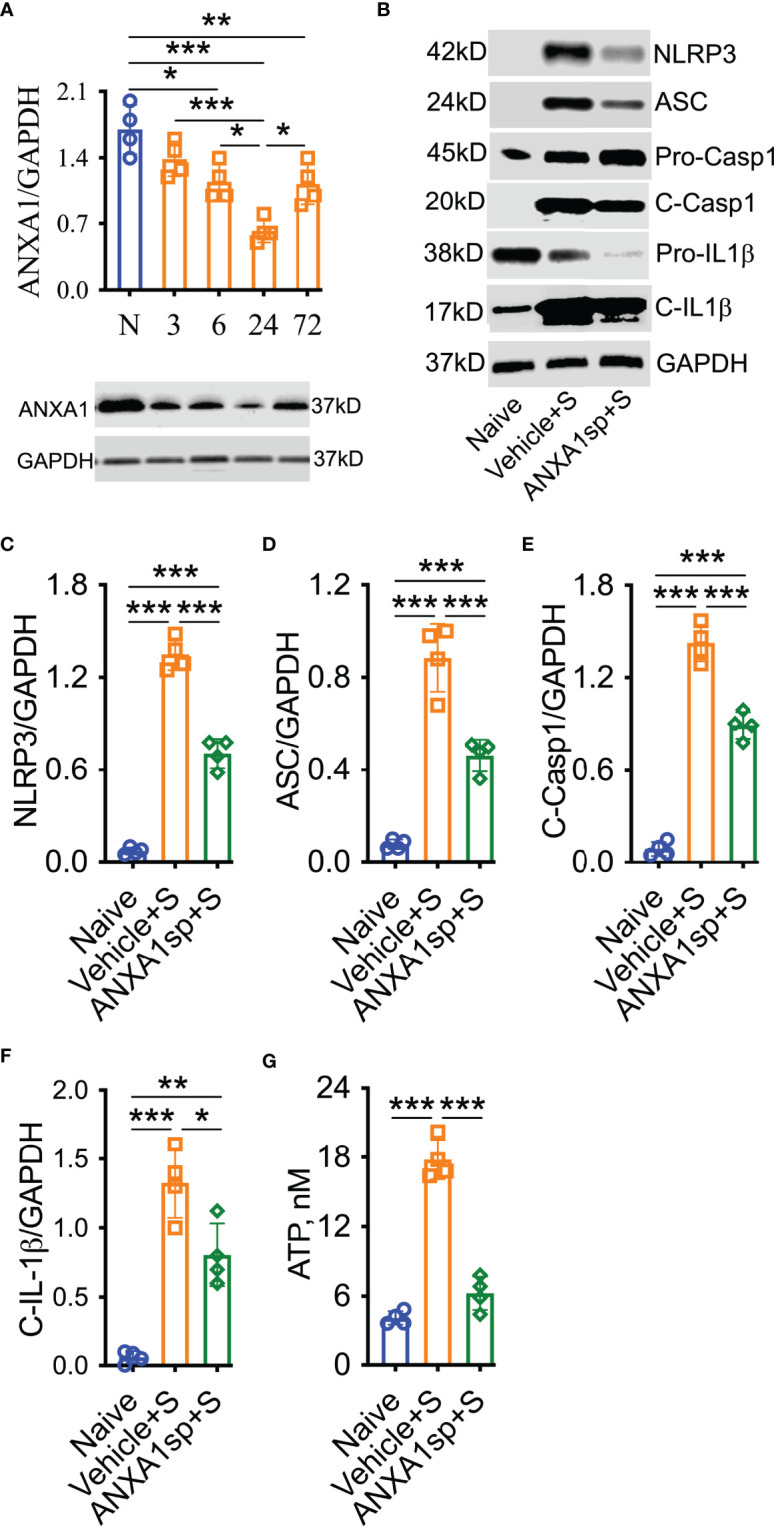
Decreased ANXA1 expression and induction of the NLRP3 inflammasome in adult mice after orthopedic surgery. Surgery reduced the expression of endogenous ANXA1 in hippocampal homogenate in a time-dependent manner, with the lowest expression found at 24-h **(A)**. Hence, we treated mice with ANXA1sp (1 mg/kg, i.p.) 30 min before surgery and evaluated NLPR3 inflammasome activation at this timepoint. Postoperative NLRP3 inflammasome activation was significantly attenuated by ANXA1sp **(B, C)**, as evidenced by Western blot analysis of ASC **(D)**, cleaved caspase-1 **(E)**, and cleaved IL-1 **(F)** in hippocampal homogenate. Surgery-induced hippocampal ATP was also reduced by ANXA1sp treatment **(G)**. Data are presented as mean ± SEM. *n* = 4/group. *P < 0.05, **P < 0.01, ***P < 0.001; analyzed by one-way ANOVA with Bonferroni test. Representative data are from two independent experiments.

Failed resolution of CNS inflammation has been proposed as a driver of this condition in PNDs (25), yet the role of ANXA1 in postoperative inflammation remains unknown. After orthopedic surgery, hippocampal expression of the endogenous pro-resolving mediator ANXA1 was significantly reduced, peaking at 24 h (0.63 ± 0.06) compared to naïve (1.7 ± 0.13, *P* < 0.001) and almost fully resolving by 72 h (1.13 ± 0.11, *P* < 0.01 vs. naïve; **Figure 1A**). Given the acute reduction in endogenous ANXA1, we next treated mice with 1 mg/kg ANXA1sp (i.p.) or vehicle (0.5% DMSO in saline, i.p.) 30 min before surgery. After orthopedic surgery, the NLRP3 inflammasome was found to be activated in the CNS of vehicle-treated animals when compared to naïve controls (1.35 ± 0.05 vs. 0.07 ± 0.01, *P* < 0.001; **Figures 1B, C****)**, including increased ASC expression (0.89 ± 0.07 vs. 0.08 ± 0.01, *P* < 0.001; **Figures 1B, D****)**, activation of caspase-1 (1.43 ± 0.06 vs. 0.09 ± 0.02, *P* < 0.001; **Figures 1B, E****)**, and cleavage of IL-1β (1.33 ± 0.13 vs. 0.06 ± 0.02, *P* < 0.001; **Figures 1B, F****)**. Notably, NLRP3 inflammasome activation was effectively reduced in ANXA1sp-treated mice (0.71 ± 0.05) compared to vehicle-treated mice after surgery (*P* < 0.001; **Figures 1B, C****)**. Hippocampal expression of ASC (0.461 ± 0.03; **Figures 1B, D****)**, caspase-1 (0.89 ± 0.04; **Figures 1B, E****)**, and IL-1β (0.81 ± 0.11; **Figures 1B, F****)** were all significantly inhibited in ANXA1sp-treated mice. Hippocampal levels of ATP were also upregulated after surgery in vehicle-treated animals compared to naïve controls (17.81 ± 0.86 vs. 4.14 ± 0.29, *P* < 0.001; **Figure 1G**). However, ATP was not induced in ANXA1sp-treated mice (6.26 ± 1.44) compared to naïve animals, while it was significantly reduced relative to vehicle-treated mice (*P* < 0.001; **Figure 1G**).

**Figure 2:**
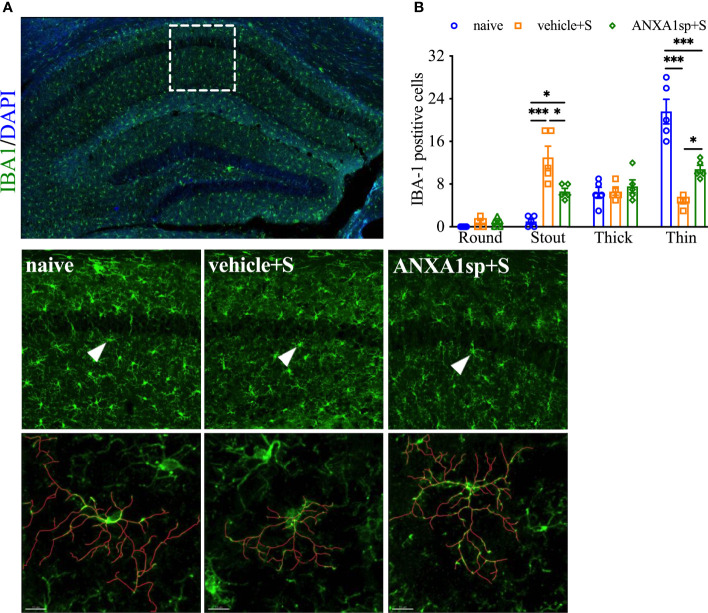
Effects of ANXA1sp on microglial morphology after surgery. Microglial morphologic changes were significantly curtailed 24 h after orthopedic surgery in adult mice pretreated with ANXA1sp **(A, B)**. Microglial morphology was quantified based on four morphologic subtypes: Round, Stout, Thick, and Thin; three representative areas/mice were used for quantification and one single microglia was represented in the reconstruction (red indicates filament tracing). Data are presented as mean ± SEM from hippocampal CA1 area. *n* = 5/group. *P < 0.05, ***P < 0.001. Analyzed by one-way ANOVA with Tukey’s multiple comparisons test. Scale bar: 20 μm. Representative data are from two independent experiments.

Morphologic changes in microglia have been reported in several models of surgery-induced cognitive impairments, including orthopedic surgery (26). Indeed, in our model of orthopedic surgery, we observed postoperative microglial morphologic changes in the hippocampus, with more stout (13.00 ± 2.1 vs. 1.00 ± 0.45, *P* < 0.001; **Figures 2A, B****)** and fewer thin Iba-1-postive cells at 24 h (4.80 ± 0.49 vs. 21.60 ± 2.32, *P* < 0.001; **Figures 2A, B****)** indicating a pathological state compared to naïve controls. Tracing of these individual microglia further showed this stout morphology by decreased branch length with increased soma size after orthopedic surgery compared to thinner cells in naïve controls. Notably, postoperative microgliosis was improved in adult mice pretreated with ANXA1sp, with Iba-1 immunoreactivity shifted toward a more resting and ramified phenotype.

### Surgery-Induced Neuroinflammation and the NLPR3 Inflammasome Activation

**Figure 3:**
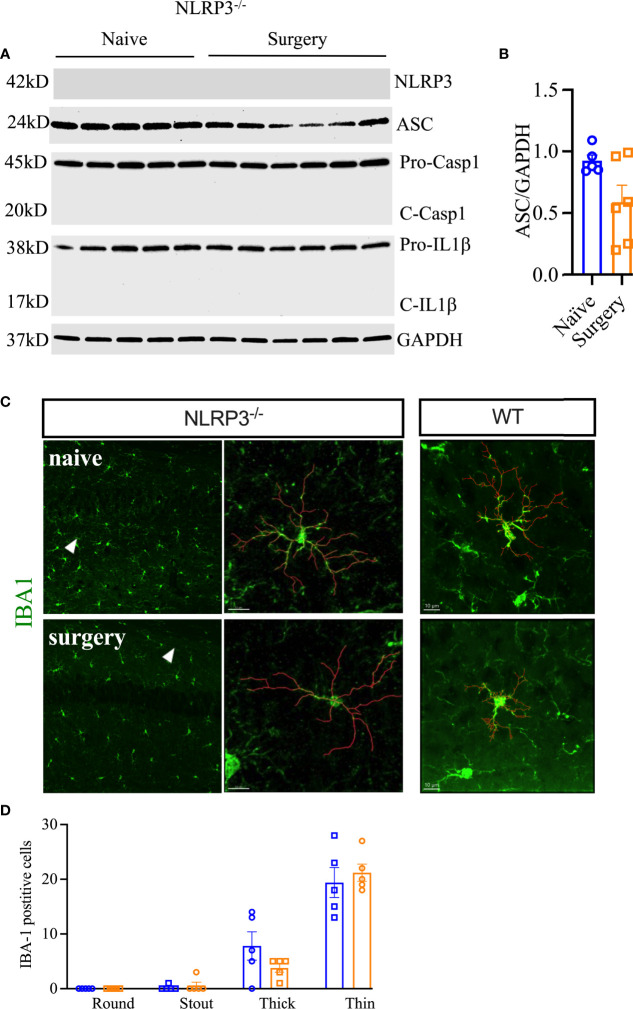
Postoperative neuroinflammation in *Nlrp3*^-/-^ mice. The NLRP3 inflammasome was not activated in the hippocampus of these animals at 24 h post orthopedic surgery **(A, B)**. Moreover, no changes in microglial morphology were observed in *Nlrp3*^-/-^ mice after orthopedic surgery, as evidenced by IBA-1 immunostaining **(C, D)**. Microglial representative filaments are provided from age matched C57BL6/J mice (WT) as comparison. For quantitation of WT data, refer to **Figure 2B**. Three representative areas/mice were used for quantification and one single microglia was represented in the reconstruction (red indicates filament tracing) from hippocampal CA1 area of *Nlrp3*^-/-^ mice only. Data are presented as mean ± SEM. *n* = 5-6/group. Scale bar: 20 μm. Representative data are from one experiment.

To confirm the role of the NLRP3 inflammasome in surgery-induced neuroinflammation, *Nlrp3*^-/-^ and *Asc*^-/-^ adult mouse lines were used. *Nlrp3*^-/-^ mice remained protected from surgery-induced neuroinflammation. In fact, compared to wild-type mice (presented above), surgery in *Nlrp3*^-/-^ mice failed to upregulate expression of ASC (0.59 ± 0.14 vs. 0.92 ± 0.05, *P* = 0.06) as shown by Western blot analysis (**Figures 3A, B****)**. Caspase-1 and cleavage of pro-IL-1β are non-detectable in *Nlrp3*^-/-^ mice. Further, *Nlrp3*^-/-^ displayed no signs of reactive microgliosis at 24 h after surgery, retaining thin and ramified morphologies (**Figures 3C, D****)**.

**Figure 4:**
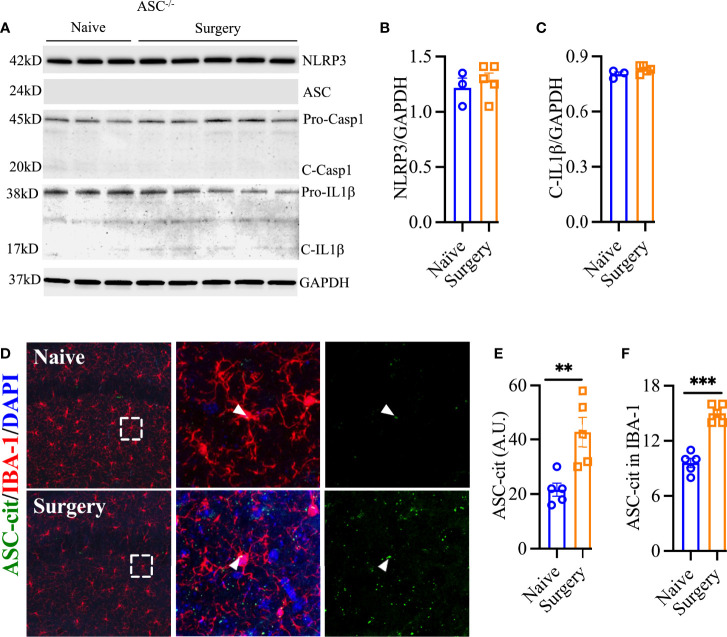
ASC speck formation after surgery. *Asc*^-/-^ showed no evidence of NLRP3 inflammasome activation in hippocampal lysate **(A–C)**. ASC-cit reporter mice showed significant speck formation in the hippocampus, including co-expression with IBA-1-positive cells **(D–F)**. Three representative areas/mice were used for quantification and one single microglia was represented in the reconstruction (red indicates filament tracing) from hippocampal CA1 area. Age matched WT controls are described in **Figures 1** and **2**. Data are presented as mean ± SEM. *n* = 3-5/group. **P < 0.01, ***P < 0.001; analyzed by two-tailed unpaired Student’s *t* test. Scale bar: 20 μm. Representative data are from one experiment.

Next, we evaluated *Asc^-/-^* mice and found protection from surgical trauma that was similar to our findings in *Nlrp3^-/-^* mice, with minimal effects on NLRP3 (1.28 ± 0.07 vs. 1.22 ± 0.09, *P* = 0.56) and cleavage of pro-IL-1β (0.83 ± 0.01 vs. 0.80 ± 0.01, *P* = 0.13; **Figures 4A–C**). To further evaluate the role of NLRP3 inflammasome activation in surgery-induced inflammation, we used ASC-citrine (ASC-cit) reporter mice. After surgery, ASC-cit mice displayed a significant increase in ASC speck formation, indicating the activated NLRP3 inflammasome complex, at 24 h (42.80 ± 5.46 vs. 21.60 ± 2.4, *P* < 0.01**;** **Figures 4D–F****)**. These mice also showed signs of hippocampal microgliosis similar to C57BL6/J mice, with greater Iba-1 immunoreactivity and more stout cell bodies 24 h after surgery. Notably, some of these ASC specks were co-localized with Iba-1-postive microglia (15.00 ± 0.4 vs. 9.60 ± 0.51, *P* < 0.001; **Figures 4D, F****)**.

### PND-Like Behavior in Adult Mice Pretreated With ANXA1sp

**Figure 5:**
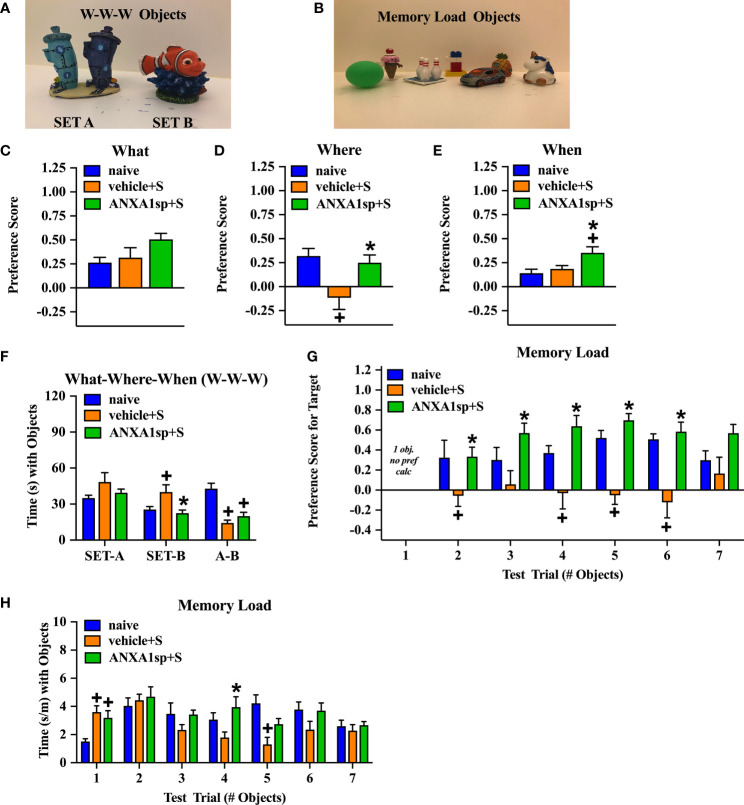
PND-like cognitive responses in adult mice treated prior to surgery with the vehicle or ANXA1sp. Example of Set A and Set B objects used in the “What-Where-When” memory task **(A)**. Example of the seven unique objects used in the Memory Load task **(B)**. Preference scores for “What” object recognition memory in the “What-Where-When” task **(C)**. Preference scores for “Where” recognition memory for the displaced object in the “What-Where-When” task **(D)**. Preference scores for “When” recognition memory for recent objects to previously investigated objects in the “What-Where-When” task **(E)**. The time (s) spent exploring Set A and Set B objects, and at testing in the “What-Where-When” task **(F)**. Preference scores for the novel object in the Memory Load test **(G)**. Mean time (adjusted by trial length) that animals explored objects in the seven consecutive trials in the Memory Load test **(H)**. N = 10 mice/treatment group for all tests. *P < 0.05, compared to vehicle + surgery mice; ^+^*P* < 0.05, compared to naïve animals. Results are shown as mean ± SEM and analyzed by MANOVA. Representative data are from one experiment.

The mice were 3 months of age at the time of behavioral testing. To study the functional effects of ANXA1sp, we evaluated cognitive responses that are affected in our PND-like model (**Figures 5A, B**). In the “What-Where-When” task (27), mice were given free access to two sets of four objects. At testing, “What” memory was examined to determine whether the animal remembered the replacement of an object with another; “Where” memory analyzed if the animals recalled where an object had been previously located; and “When” memory reflected whether the mouse remembered the objects that were last investigated. For “What” memory, responses among the three groups (naïve, surgery plus vehicle injection, and surgery plus ANXA1sp treatment) were not statistically distinguished (**Figure 5C**; **Table 1**). Nevertheless, marked statistical differences in object preferences for the “Where” (**Figure 5D**) and “When” (**Figure 5E**) memory tests were evident (**Table 1**). For “Where” memory the vehicle-treated mice with surgery showed a significant reduction in their abilities to identify the displaced object compared to the naïve control (*P*<0.011) and mice given ANXA1sp prior to surgery (*P*<0.038); these two latter groups had similar preferences for the displaced object. The “When” comparison was more difficult for the animals. Both the naïve and vehicle + surgery mice had similar moderate preference scores. By contrast, mice treated with ANXA1sp prior to surgery had significantly higher preference scores than the other two groups (*P*-values<0.052). The higher preference scores for the ANXA1sp + surgery mice cannot be attributed to longer times spent with the objects or differential locomotor activities since they had some of the lowest object interaction times across training and testing than any other group (**Table 2**). Indeed, no significant group differences in object interaction times were discerned at training with the set A objects (**Figure 5F**). At training with set B objects, mice treated with the vehicle prior to surgery spent more time interacting with the objects compared to the naïve mice and those given ANXA1sp prior to surgery (*P*-values<0.051). Interestingly, at testing the vehicle + surgery and ANXA1sp + surgery mice spent less time exploring objects compared to the naïve animals (*P*-values<0.001).

In the memory load test naïve mice and those treated with ANXA1sp prior to surgery demonstrated an ability to identify the novel object even as the memory load increased with up to six objects (**Figure 5G**; **Table 3**). By comparison, the mice treated with the vehicle prior to surgery were impaired. Here, the vehicle + surgery mice had significantly reduced preferences for the novel object compared to naïve group when 2 (*P*<0.052), 4 (*P*<0.051), 5 (*P*<0.001), and 6 (*P*<0.001) objects were present. Although performance by the mice in the naïve and ANXA1sp + surgery groups were similar, the ANXA1sp-treated group performed even better than the vehicle + surgery mice when 2 (*P*<0.057), 3 (*P*<0.018), 4 (*P*<0.001), 5 (*P*<0.001), and 6 (*P*<0.001) objects were presented. When time spent investigating objects across trials was examined, few group differences were observed (**Figure 5H**; **Table 4**). However, on the first trial both treatment groups with surgery engaged in higher object exploration times than the naïve control (*P*-values<0.022). When 4 objects were present, mice in the ANXA1sp + surgery group spent more time in object exploration than naïve mice (*P*<0.032). With 5 objects the naïve animals interacted with the objects for a longer time than the vehicle + surgery mice (*P*<0.001). Together these results demonstrate that vehicle-treated tibia-fracture mice are markedly impaired in cognitive tasks compared to naïve mice, and that treatment with ANXA1sp prior to surgery prevents these cognitive impairments.

### Postoperative Neuroinflammation in Aged Mice Treated With ANXA1sp

**Figure 6:**
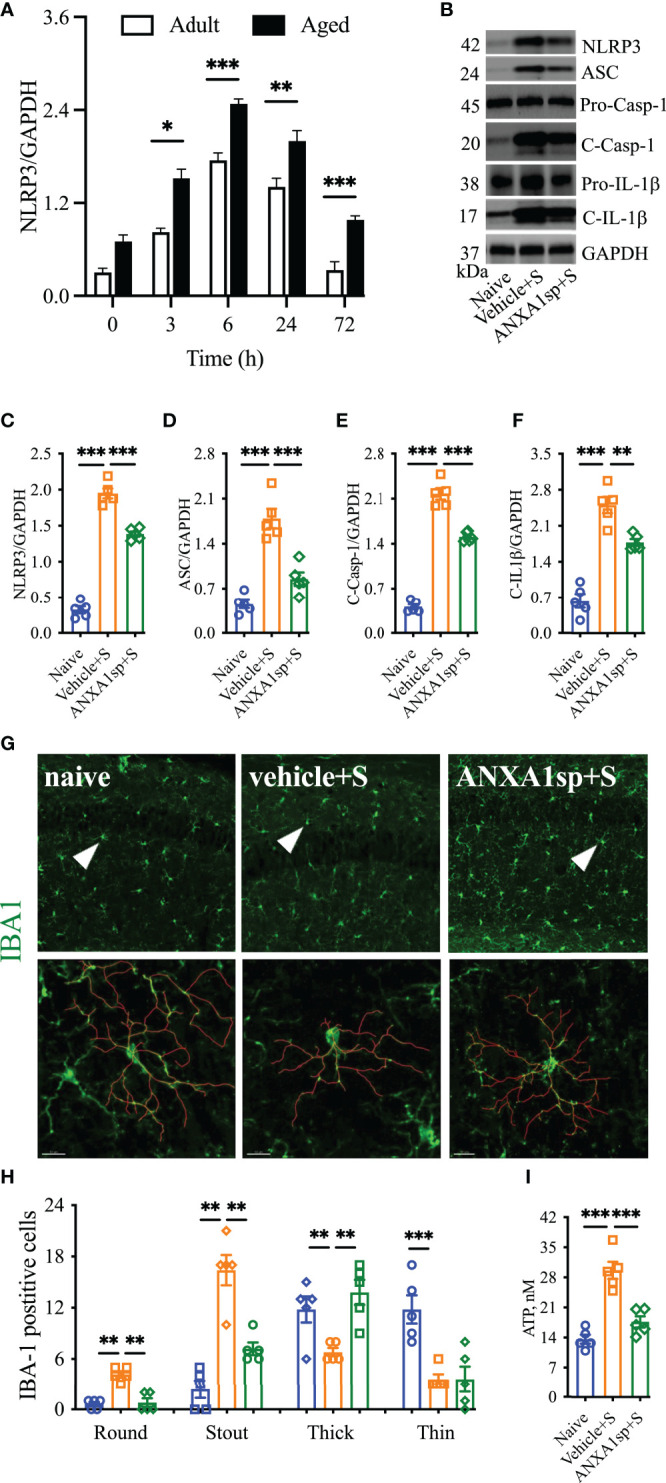
Effects of ANXA1sp on NLRP3 inflammasome and microglial morphology in aged mice after surgery. The hippocampal NLRP3 inflammasome complex was significantly activated in a time-dependent manner in aged mice after orthopedic surgery, with older mice showing a significantly higher baseline compared to adult mice **(A)**. Hippocampal NLRP3 inflammasome activation **(B–F)** was attenuated in aged mice treated with ANXA1sp (1 mg/kg, ip) 30 min before orthopedic surgery (same dose and timepoint as in the adult mice). Similarly, microglial activation in the CA1 area was reduced 24 h after surgery **(G, H)**, especially improving ameboidal morphologies (round, stout, and think). Three representative areas/mice were used for quantification and one single microglia was represented in the reconstruction (red indicates filament tracing). Hippocampal ATP expression **(I)** was also restored in aged mice treated with ANXA1sp. Data are presented as mean ± SEM. *n* = 5. **P < 0.01, ***P < 0.001, compared to naïve or vehicle controls. Analyzed by one-way ANOVA with Tukey’s multiple comparisons test. Scale bar: 20 μm. Representative data are from two independent experiments. *P < 0.05.

Aging is a critical risk factor for many neurologic conditions, including PND (28). Aged mice showed a heightened, but similar, curve for NLRP3 activation after surgery, which peaked at 6 h (2.48 ± 0.06, *P* < 0.0001), remained elevated at 24 h (2.00 ± 0.14, *P* < 0.0001), and resolved to baseline by 72 h (0.98 ± 0.05, *P* = 0.28) compared to adult mice (0.70 ± 0.09). Notably, more NLRP3 activation was found in aged mice compared to adult mice after surgery [R^2^ = 0.98; F(1,3) = 130.5]; *P* = 0.0014; **Figure 6A**].

We next administered the same therapeutic regimen of ANXA1sp in 18- to 22-month-old C57BL6/J mice. Western blot analysis showed increased levels of NLRP3 in the hippocampus (1.95 ± 0.16 vs. 0.32 ± 0.11, *P* < 0.001; **Figures 6B–F**). This exacerbated the NLRP3 inflammasome activation which was significantly blunted in aged mice pretreated with ANXA1sp (1.39 ± 0.08 vs. 1.95 ± 0.16, *P* < 0.001; **Figure 6C**), affecting the expression of ASC (ANXA1sp vs. vehicle: 0.85 ± 0.23 vs. 1.79 ± 0.34, *P* < 0.001; **Figure 6D**), activation of caspase-1 (ANXA1sp vs. vehicle: 1.51 ± 0.07 vs. 2.18 ± 0.20, *P* < 0.001; **Figure 6E**), and levels of IL-1β (ANXA1sp vs. vehicle: 1.77 ± 0.15 vs. 2.50 ± 0.36, *P* < 0.001; **Figure 6F****)**. We next evaluated microglial activation at 24 h after surgery. Surgery triggered more stout (16.4 ± 1.78 vs. 2.4 ± 1.03, *P* < 0.001) and round (4.20 ± 0.37 vs. 0.60 ± 0.24, *P* < 0.001) Iba-1-postive cells in the hippocampus with reduced branch length compared to naïve controls. The round microglia were hardly visible in adult mice at the same time point (**Figures 6G, H****)**. Further, hippocampal ATP was retained at baseline levels in aged mice pretreated with ANXA1sp vs. vehicle (17.56 ± 103 vs. 29.56 ± 2.02, *P* < 0.001; **Figure 6I**). Overall, aged mice pretreated with ANXA1sp showed significantly improved microglial pathology, especially soma enlargement and swelling, 2 key features of CNS dyshomeostasis (29).

## Discussion

Here, we studied the relationship between resolution agonists, particularly ANXA1 signaling, and NLRP3 inflammasome activation in mediating postoperative neuroinflammation and PND-like behavior. We found that preoperative treatment with a small peptide derived from the N-terminal region of human ANXA1 (ANXA1sp) attenuates NLRP3 inflammasome activation in adult mice after orthopedic surgery, and thereby reduces microgliosis and behavioral deficits. Notably, these anti-neuroinflammatory effects were also observed in aged mice, representing a potential next step toward translating resolution agonists into perioperative clinical trials.

Dysregulated immunity has become a key pathologic hallmark of virtually every neurologic disorder including PNDs. Surgical trauma triggers a systemic inflammatory response that can impair microglial function, and thus contribute to sickness behavior and memory decline. This postoperative cytokine storm has been well documented across several rodent models as well as in surgery patients. In our earlier work, we discovered a role for IL-1β signaling in mediating surgery-induced neuroinflammation. Mice treated with IL-1 receptor antagonist (IL-1RA) or lacking IL-1R showed no signs of neuroinflammation or microglial activation (7). Subsequent studies in rats after abdominal surgery further defined the importance of *central* IL-1β signaling in contributing to PND-like behavior. In fact, only intracisternal administration of IL-1RA was effective in reducing behavioral impairments and neuroinflammation after surgical trauma (30). Studies in older adults with delirium after hip fracture repair confirmed the presence of high levels of IL-1β in cerebrospinal fluid (CSF), suggesting that IL-1 may represent a valuable biomarker for identifying and possibly treating patients at risk for PNDs (18). Indeed, IL-1β signaling has been described as a key mediator of sickness behavior (fever, HPA axis activation, and depression) (31) as well as microglial priming during aging, and overall neuroinflammation [reviewed in (32)]. However, despite seminal contributions in this field, little is known about the regulation of mature IL-1β in PNDs.

IL-1β maturation is critically regulated by the NOD-like receptor protein 3 (NLRP3) inflammasome. Canonical activation of the NLRP3 inflammasome involves recruitment of adapter proteins, including apoptosis-associated speck-like protein containing a caspase recruitment domain (ASC), and caspase-1, which orchestrate the maturation and secretion of IL-1β and IL-18 (33). Canonically, generation of mature IL-1β requires 2 signals, the production of pro-IL-1β by activating transcription factors, such as NF-κB, and maturation of IL-1β and its release by pyroptosis induced by NLRP3 inflammasome activation, stimulated by factors such as ATP (34).

**Figure 7:**
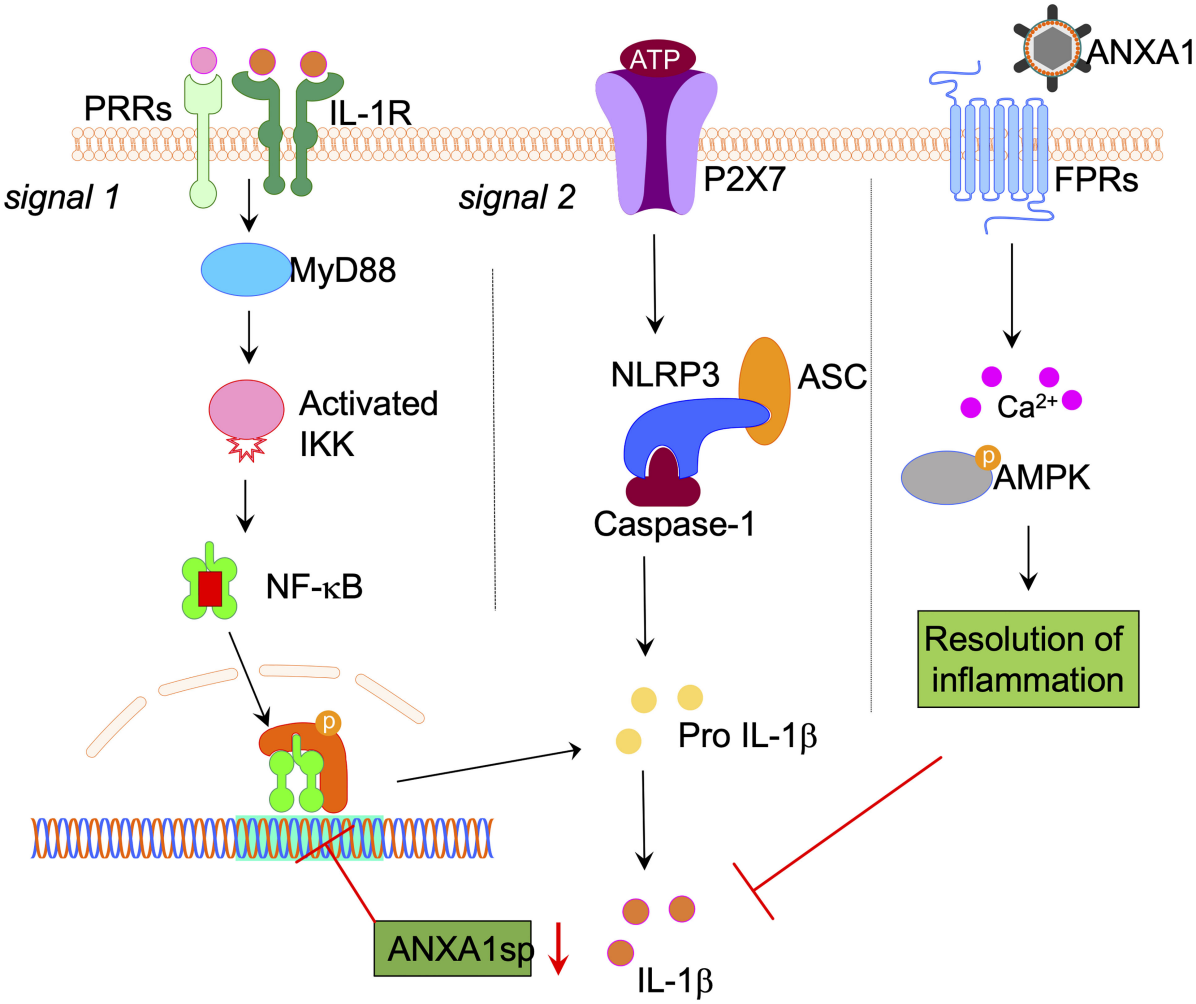
Proposed NLPR3 signaling mechanism in postoperative neuroinflammation. Aseptic surgical trauma induces a cytokine storm that engages several pattern recognition receptors (PRRs), including the interlukin-1 (IL-1) receptor. Downstream signaling from these receptors relies on adaptor proteins such as MyD88, which eventually activates NF-κB to perpetuate the inflammatory response. Parallel to this process, surgery also elevated levels of ATP in the hippocampus. In combination with NF-κB activation this induced the NLRP3 inflammasome and ASC specks formation in microglia, thus contributing to postoperative neuroinflammation. Surgery also impaired the expression of hippocampal Annexin-A1 (ANXA1) that is critically involved in inflammation-resolution and can be regulated through our biomimetic peptide. Within this framework we found that treatment with ANXA1sp regulated NLRP3 expression and levels of IL-1β postoperatively. Given the established elevation of IL-1β in preclinical and clinical biofluids of subjects with postoperative neurocognitive disorders, harnessing this pathway may provide safer strategies to treat ensuing neuroinflammation.

Surgery triggers NF-κB activation *via* multiple pathways including danger-associated molecular patterns (DAMPs), released during the acute-phase response following aseptic surgical trauma. DAMPs, such as HMGB1, are potent activators of NF-κB and the subsequent cytokine storm, which has been well described in this model of orthopedic surgery (8, 9, 35). In fact, disabling NF-κB activation in this model prevents microglial activation, further highlighting the critical role of this gene in regulating the innate immune response to surgery (36). Absence of key immune receptors, such as MyD88 and TLR4, prevents cytokine release and surgery-induced microglial activation (8, 37). In the current study, we found that NLRP3 and ASC knock-out mice remained protected from postoperative neuroinflammation, indicating an upstream mechanism for NF-κB activation. Treatment with ANXA1sp downregulated the expression of key inflammasome components, including ASC and NLRP3, and levels of IL-1β after surgery in both adult and aged mice (**Figure 7**).

Notably, we found evidence of NLRP3 inflammasome activation in microglia after surgery with formation of “ASC specks”, which result from recruitment and polymerization of ASC into ∼1-µm structures (38). Specks are not unique to activation of the NLRP3 inflammasome, but also occur with activation of NLRP1b, NLRC4, and AIM2 (39). The elevated ATP that we measured in whole hippocampal homogenate after surgery may be responsible for the microglial response, thus leading to speck formation. Indeed, extracellular ATP is a well-known activator of microglial processes after injury (40) and an activator of the NLRP3 inflammasome, and surgery may hijack their neuroprotective functions leading to a neurotoxic environment and PNDs. Additional work using more detailed approaches is needed to further characterize the microglial response and the signaling events following peripheral surgery.

Previous work described a key role for microglial activation in PNDs. PET imaging provides evidence of microgliosis in postsurgical patients (41). Here, we found that preoperative administration of ANXA1sp reduced morphologic changes associated with microglial activation in the hippocampus. These effects may result from direct modulation of resident microglia since ANXA1 is expressed on these cells, and the small peptide can easily cross the blood-brain barrier (BBB) (14). Although we did not evaluate BBB opening in the present study, it is important to note that orthopedic surgery impairs BBB integrity and the expression of tight junctions such as claudin-5 and aquaporin-1, thus facilitating infiltration of peripherally injected compounds into the CNS (35, 42). Indeed, this breach also allows systemic factors such as IgG and fibrinogen as well as inflammatory myeloid cells such as macrophages to enter the brain parenchyma and contribute to neuroinflammation (21, 36). Preventing infiltration of CCR2-positive monocytes after surgery reduces microgliosis and PND-like behavior (43). Given that ANXA1 is a key regulator of endothelial function and BBB integrity, it is plausible that these neuroprotective effects are mediated by limiting leukocyte trafficking at the neurovascular unit (44). In fact, ANXA1 peptidomimetics such as Ac2-26 exert potent effects on the vasculature by limiting neutrophil adhesion and emigration, but without impairing cell rolling (45). The ANXA1 is also highly expressed on macrophages, facilitating efferocytosis of apoptotic neutrophils and overall resolution of inflammation (46, 47). The pro-resolving effects of ANXA1sp may target this critical step at the level of the BBB, although our peptide also exerts potent anti-inflammatory effects on systemic cytokines (16). Thus, ANXA1sp effects on the CNS may result from an overall dampening of the pro-inflammatory milieu rather than, or in addition to, a direct effect on the BBB.

ANXA1 is a 37-kDA protein that is a member of a larger annexin superfamily of calcium-dependent phospholipid-binding proteins that signal *via* G protein-coupled receptors and formyl peptide receptor 2 (FPR2) (48), thus inhibiting phospholipase A_2_ (PLA_2_) and downstream production of bioactive lipid mediators derived from arachidonic acid release (49). Notably, the tripeptide sequence of ANXA1sp (Ac-Gln-Ala-Trp) at the N-terminus represents a critical domain for signaling *via* FPR1 and exerts anti-inflammatory effects (50). Indeed, FPR1 signaling is important in activating the NLRP3 inflammasome as evidenced by reduced inflammation, leukocyte infiltration, and tissue damage in *Fpr-1* KO mice (51), as well as limiting neuroinflammation following stroke (52). Recent work by Galvão et al. demonstrated that AnxA1 co-localizes and directly binds to the NLRP3 (53), thus these anti-inflammatory effects may involve several pathways in addition to FPR signaling including modulation of neutrophils and macrophages (54, 55). ANXA1 also facilitates resolution of inflammation by acting on the lipoxin A4 receptor (ALXR) to downregulate polymorphonuclear neutrophil recruitment at the site of inflammation (56). These actions are important because they limit the synthesis of eicosanoids such as prostaglandins, thromboxanes, prostacyclins, and leukotrienes, which are upregulated in the CNS of patients after peripheral surgery (57). ANXA1 signaling *via* ALXR was recently shown to support regeneration of skeletal muscle after injury (58), which may translate to the surgical setting. These regulatory effects of ANXA1 and many other pro-resolving mediators provide unique opportunities to apply resolution pharmacology to the clinical setting, given their known safety profile.

In summary, we have found a previously unrecognized action of an ANXA1 peptidomimetic on the NLRP3 inflammasome complex, which resulted in reduced microglial activity and PND-like behavior after surgery. Although further work is needed to determine the specific site of action for ANXA1sp, our findings provide a novel target for regulating postoperative neuroinflammation and cognitive disorders during aging.

## Data Availability Statement

The raw data supporting the conclusions of this article will be made available by the authors, without undue reservation.

## Ethics Statement

All procedures were conducted under protocols approved by the Institutional Animal Care and Use Committee at Duke University in accordance with the guidelines of the National Institutes of Health for animal care (Guide for the Care and Use of Laboratory Animals, Health and Human Services, National Institutes of Health Publication No. 86-23, revised 1996).

## Author Contributions

ZZ and QM performed experiments and analyses. RV performed confocal microscopy and imaging analyses. WB generated the transgenic mice and contributed to the interpretation of the data. WW and RR designed, conducted, and analyzed the behavioral experiments. TY and MS provided analytical tools and reagents. ZZ, MS, and NT designed the study. All authors contributed to the writing. All authors contributed to the article and approved the submitted version.

## Funding

This work was in part supported by the National Institutes of Health (NIH) R01AG057525, a School of Medicine voucher for studies conducted in the Mouse Behavioral and Neuroendocrine Analysis Core Facility, and Duke Anesthesiology Departmental funds. Some of the behavioral experiments were conducted with equipment and software purchased with a North Carolina Biotechnology Center grant.

## Conflict of Interest

ZZ and QM are co-inventors on patents filed through Duke University on the therapeutic use of ANXA1sp.

The remaining authors declare that the research was conducted in the absence of any commercial or financial relationships that could be construed as a potential conflict of interest.

## Publisher’s Note

All claims expressed in this article are solely those of the authors and do not necessarily represent those of their affiliated organizations, or those of the publisher, the editors and the reviewers. Any product that may be evaluated in this article, or claim that may be made by its manufacturer, is not guaranteed or endorsed by the publisher.
